# CUGBP1, a crucial factor for heart regeneration in mice

**DOI:** 10.1038/s41419-022-04570-w

**Published:** 2022-02-08

**Authors:** Yang Liu, Huiwen Wang, Han Zhang, Jun Wang, Qun Liu, Youkun Bi, Shaole Song, Xinlong Qiao, Keqi Zhu, Yanyun Wu, Guangju Ji

**Affiliations:** 1 Institute of Biophysics, Chinese Academy of Sciences, 100101 Beijing, China; 2 Pediatric Cardiac Center, Beijing Anzhen Hospital, Capital Medical University, 100029 Beijing, China

**Keywords:** Cell proliferation, Self-renewal

## Abstract

The mammalian heart is capable of achieving perfect regeneration following cardiac injury through sustained cardiomyocyte proliferation during the early period after birth. However, this regenerative capacity is lost by postnatal day 7 and throughout adulthood. CUGBP1 is critical for normal cardiac development but its role in heart regeneration remains unclear. Cardiac CUGBP1 levels are high in the early postnatal period and soon downregulate to adult levels within 1 week following birth in mice. The simultaneously diminished regenerative capacity and CUGBP1 levels by postnatal day lead us to hypothesize that CUGBP1 may be beneficial in heart regeneration. In this study, the function of CUGBP1 in heart regeneration was tested by a heart apex resection mouse model. We demonstrate that cardiac inactivation of CUGBP1 impairs neonatal heart regeneration at P1, in turn, replenishment of CUGBP1 levels prolong regenerative potential at P8 and P14. Furthermore, our results imply that the Wnt/β-catenin signaling and GATA4 involve in the CUGBP1 modulated neonatal heart regeneration. Altogether, our findings support CUGBP1 as a key factor promoting post-injury heart regeneration and provide a potential therapeutic method for heart disease.

## Introduction

The adult mammalian heart has limited capacity for regeneration or repair, which is insufficient to restore contractile function following injury and disease [1, 2]. Thus, the loss of cardiomyocytes results in cardiac dysfunction, which ultimately contributes to heart failure, a primary cause of human mortality worldwide. In contrast, zebrafish shows a high capacity for heart regeneration throughout life and is capable of full recovery from significant injury [3]. Similarly, the neonatal mouse heart retains significant cardiac regenerative potential after apical resection [4]. However, the regenerative response is lost within 7 days after birth and remains absent in adult hearts, which is replaced by a fibrotic response and pathological hypertrophy [4]. Therefore, it is crucial to understand the molecular mechanisms that regulate cardiac regenerative capacity in mammals and extending the time window of mammalian heart regeneration, which represents an important issue for cardiovascular regenerative medicine.

CUG triplet repeat RNA binding protein 1 (CUGBP1) is a member of the CUGBP Elav-like family (CELF), which plays an important role in cardiac development and function [5]. Cardiac CUGBP1 levels are high in the early postnatal period and soon downregulate to adult levels within 1 week following birth in mice [6]. The simultaneously diminished regenerative capacity and CUGBP1 levels by postnatal day lead us to speculate that CUGBP1 may be beneficial in heart regeneration. However, the regulatory role of CUGBP1 in heart repair and regeneration remains unknown.

The Wnt signaling pathways are a group of signal transduction pathways, which begin with proteins that pass signals into a cell through cell surface receptors. Wnt signaling pathways are activated by the binding of a Wnt-protein ligand to a frizzled family receptor. Wnt signaling was characterized for its role in embryonic development, including body axis patterning, cell fate specification, cell proliferation and cell migration. These processes are necessary for proper formation of important tissues including heart. Previous research identified that the Wnt signaling responsible for cardiomyocyte proliferation and cardiac regeneration [7, 8].

In this study, the function of CUGBP1 in heart regeneration was tested by a heart apex resection mouse model. We demonstrate that cardiac inactivation of CUGBP1 impairs neonatal heart regeneration, in turn, replenishment of CUGBP1 levels prolongs regenerative potential of mice up to 14 days after birth. Our finding provides novel molecular pathway involved in the heart regeneration.

## Results

### CUGBP1 abundance is upregulated upon apical resection

In order to investigate the expression pattern of CUGBP1 in the process of heart regeneration in newborn mice, apical resection was performed on 1-day-old (P1) and 8-day-old (P8) mice.

**Fig. 1:**
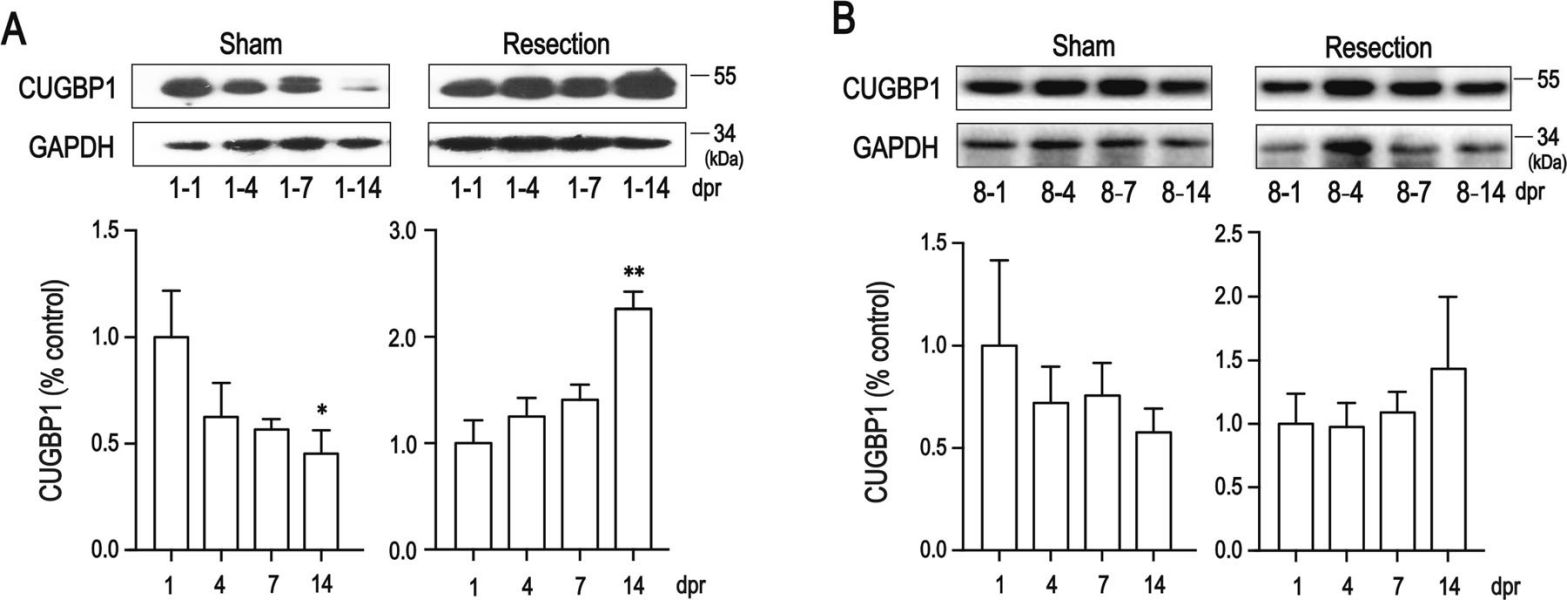
CUGBP1 abundance is upregulated after apical resection. One-day (**A**) or 8-day (**B**) old mice were performed sham or apical resection surgery, CUGBP1 levels in cardiac tissue samples were detected by western blot analysis at 1,4,7,14 days post resection. Western blot bands were quantified by densitometry and normalized to GAPDH. dpr, days post resection. Data are the mean ± SEM (*n* = 4–6). *p < 0.05, **p < 0.01 vs. control.

As shown in Fig. 1, the CUGBP1 abundance decreased gradually in the sham group, while in 1-day-old apical resection group, which is capable of regeneration, the CUGBP1 levels remained high, with a gradual upward trend, up to 2.3 times at P15 (Fig. 1A). Whereas, there was no significant change in the CUGBP1 expression after apical resection in 8-day-old unrepairable mouse hearts (Fig. 1B). These results suggest that CUGBP1 may play a role in regeneration of heart injury in newborn mice.

### CUGBP1 is necessary for neonatal heart regeneration

Genetically CUGBP1 modified mice display a number of overt illness, including cardiac abnormalities [5, 9, 10]. To overcome the disadvantage, CUGBP1 levels were manipulated in the neonatal mouse heart by recombinant adenoviral gene transfer in our study. Seven days after injection, the expression of DsRed tagged exogenous protein in the heart of mice was observed by fluorescence microscopy. The red fluorescence signal is the DsRed signal expressed externally (Supplementary Fig. S1A, B). Importantly, none persistent impairments of the heart were observed post injection (Supplementary Fig. S1C, D).

**Fig. 2:**
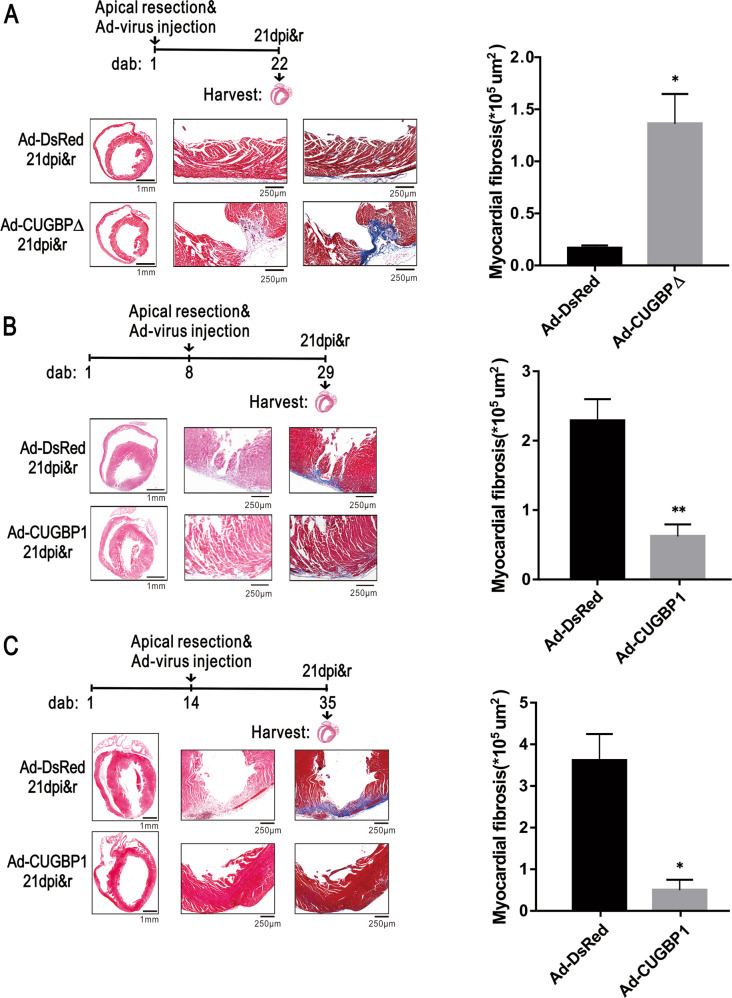
CUGBP1 reconstitution markedly promoted heart regeneration. One-day (**A**), 8-day (**B**), or 14-day (**C**) old mice were administrated with Ad-virus and apical resection was performed simultaneously. The HE staining of the whole heart and HE, Masson trichromatic staining of the apex of the heart at high magnification were presented (*n* = 3–4). dab, days after birth; dpi&r, days post injection & resection. Statistical analysis of the myocardial fibrosis are presented in the right panel.

To directly assess the impact of CUGBP1 on heart regeneration, tissue paraffin sections were stained with HE and Masson’s trichrome staining of mouse hearts after apex resection. As shown in Fig. 2A, apical resection using 1-day-old mice, the control group hearts achieved full restoration, while the regeneration of the CUGBP∆ (inactivation of CUGBP1 [11]) heart was replaced by an increase in myocardial tissue fibrosis (Fig. 2A). In turn, using 8-day-old and 14-day-old mice, the hearts of control group failed to regenerate, instead, scar repair took place. Impressively, CUGBP1 reconstitution markedly promoted heart regeneration at P8 (Fig. 2B) and P14 (Fig. 2C). These results suggest that CUGBP1 levels are important in the heart regeneration following apical injury.

### CUGBP1 promotes cardiomyocyte proliferation in apical injury heart

**Fig. 3:**
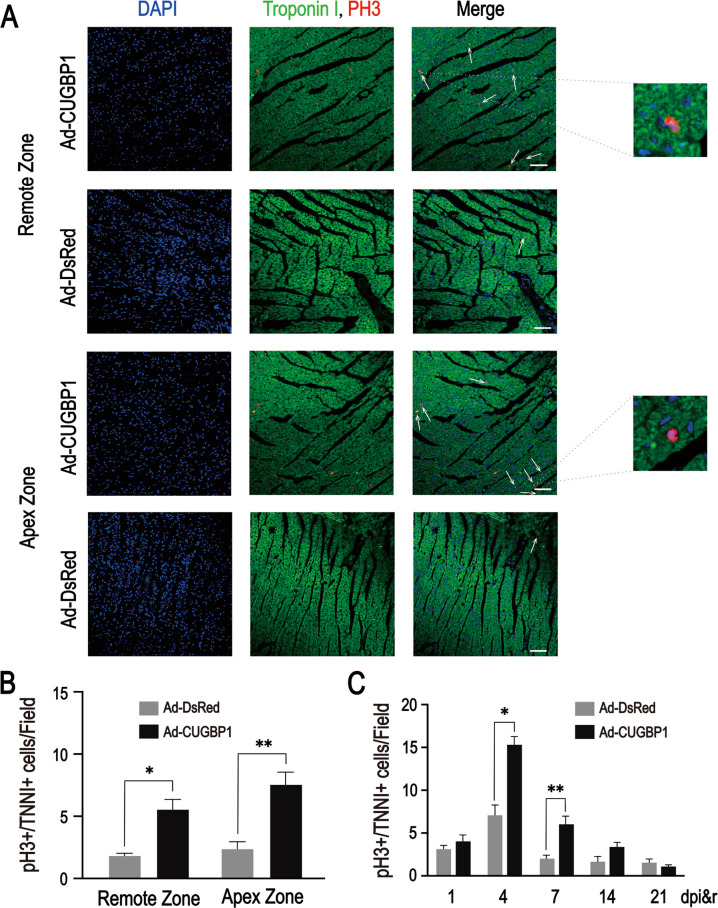
CUGBP1 promotes cardiomyocyte proliferation in apical resection mice heart. **A** CUGBP1 was overexpressed in the 14-day old mice hearts and apical resection was performed simultaneously. Seven days after resection, cardiac tissue from apex zone was collected for paraffin section immunofluorescence detection of PH3 (red) and TNNI (green). Representative images are presented. **B** Statistical analysis of the number of PH3/TNNI-positive cells in apex and remote zone in the 14-day old mice heart following the Ad-virus infection and resection on seven days after resection. **C** Statistical analysis of the number of PH3/TNNI-positive cells by paraffin sections for immunofluorescence in the 14-day old mice heart following the Ad-virus infection and resection. Scale bars: 50 μm, 10 μm(inset). Data are presented as the mean ± SEM (*n* = 8, 9 fields each mouse). *p < 0.05, **p < 0.01 vs. control. dpi&r, days post injection & resection.

Cardiomyocyte proliferation is a major cellular mechanism driving heart regeneration in neonatal mice [4] and adult zebrafish [12]. The proliferation of cardiomyocytes was detected by immunofluorescence staining with Phosphorylated histone H3 (PH3, a cell proliferation marker) and Troponin I (a cardiomyocyte marker). As shown in Fig. 3, PH3-positive signals (red signal) were obviously visible in the apex and remote zone of the mouse hearts and co-localized with Troponin I (green signals). PH3-positive signals were evident in CUGBP1 replenishment group in the apex and remote zone of the mouse hearts (Fig. 3A, B). Similarly, the pH3-positive cells in CUGBP1 replenishment group were significantly higher than that in the control from the hearts of mice in 4, 7 days after apical resection (Fig. 3C). These results indicated that CUGBP1 promotes cardiomyocyte proliferation in heart of mice after injury.

### CUGBP1 mediates cardiomyocyte proliferation through Wnt/β-catenin signaling

**Fig. 4:**
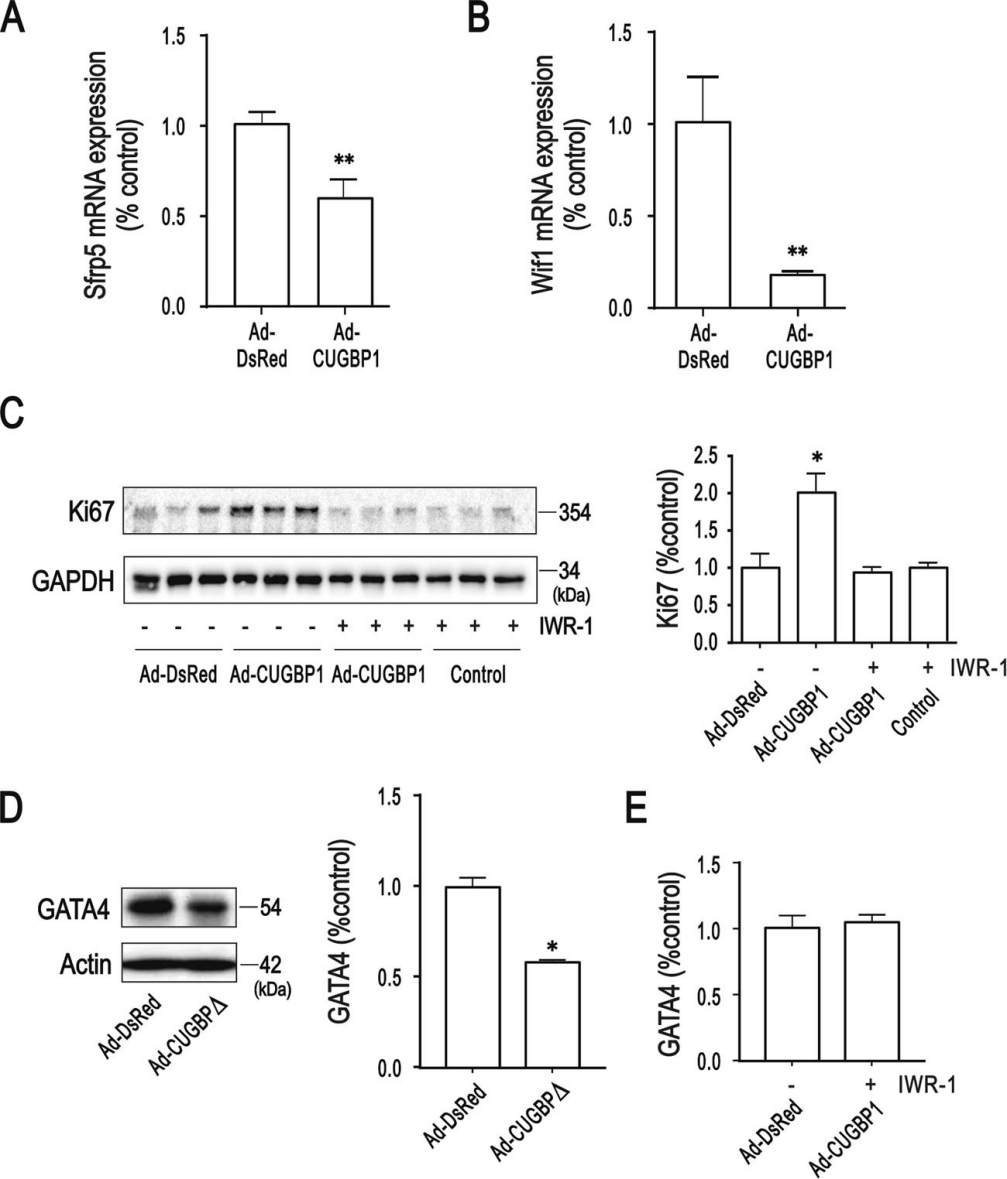
CUGBP1 mediates cardiomyocyte proliferation through Wnt /β-catenin pathway. **A**, **B** Cardiac tissues from 14-day old mice with CUGBP1 over-expression and apical resection were collected at 7 days after the surgery and mRNA expression level of Sfrp5 and Wif1 were determined by real-time quantitative PCR. **C** Western blotting of ki67 in Ad-virus infected primary cardiomyocyte with IMR treatment. Bands were quantified by densitometry and normalized to GAPDH. **D** Statistical analysis of western blot with GATA4 expression upon CUGBP1 inactivation in 1-day old mice heart. **E** Statistical analysis of western blot with GATA4 abundance upon CUGBP1 over-expression and IWR-1 treatment. Data are the mean ± SEM (*n* = 3). *p < 0.05, **p < 0.01 vs. control.

Next, we sought to understand the mechanisms underlying CUGBP1 promoted neonatal heart regeneration. Transcriptome sequencing was performed by using hearts at 7 d after apical resection upon CUGBP1 over-expression from mice (Supplementary Fig. S2). In support of transcriptome data and the qPCR, we found that Sfrp5 and Wif1, Wnt/β-catenin inhibitory factors, were significantly downregulated upon CUGBP1 over-expression (Fig. 4A, B). Furthermore, by using IWR-1, a selective Wnt/β-catenin signaling inhibitor, treatment for 8 h in neonatal rat ventricular myocytes (NRVMs), the increased Ki67 levels upon CUGBP1 over-expression was significantly reduced (Fig. 4C). Therefore, we speculate that CUGBP1 can promote cardiomyocyte proliferation through the activation of Wnt/β-catenin signal pathway.

Interestingly, GATA4, a critical cardiac factor for neonatal heart regeneration, demonstrates parallel expression profile with the CUGBP1 during the postnatal period [13]. Inactive CUGBP1 resulted in significantly diminished GATA levels at P1 by immunoblotting (Fig. 4D). In contrast, we show that IWR-1 treatment dismissed CUGBP1-dependent regulation of GATA4 (Fig. 4E).

**Fig. 5:**
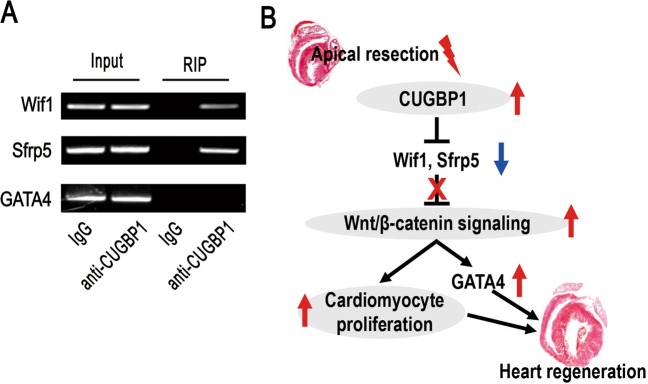
CUGBP1 directly binds to the RNA of Wif1 and Sfrp5. **A** RIP analysis was carried out using antibodies against CUGBP1 in NRVMs and RT-PCR assay was performed using primers targeting Wif1, Sfrp5, or GATA4, respectively. **B** Proposed mechanism of CUGBP1-Wnt/β-catenin-GATA4 regulatory axis. CUGBP1 abundance is upregulated upon apical resection. Therefore, altered levels of CUGBP1 interfere and downregulate the Sfrp5 and Wif1, which leads to activation of Wnt/β-catenin signaling. Consequently, cardiomyocyte proliferation and GATA4 levels is elevated, which could contribute to heart regeneration.

By binding to RNAs, CUGBP1 direct a variety of regulatory events [14]. We next explored whether CUGBP1 directly binding to RNA of Wif1, Sfrp5 or GATA4. The physical association of CUGBP1 and RNAs was examined by RNA immunoprecipitation (RIP) using NRVMs. The coprecipitated RNA was subjected to RT-PCR to demonstrate the presence of Wif1, Sfrp5 or GATA4 RNAs. Our results indicate that CUGBP1 specially bond to Wif1 and Sfrp5 RNA, but not GATA4 (Fig. 5A).

## Discussion

### CUGBP1 is crucial for heart regeneration

The mechanism of the postnatal heart regeneration switch from fetal to adult is largely unknown. To date, several studies on mammalian indicate that murine heart lost regeneration capability at postnatal day 7 [2, 4, 13]. In the present study, we, for the first time, extends the time window of the mammalian heart regeneration ability up to 14 days after birth. CUGBP1, an important regulator in heart, protein levels begin to decrease by P6 [6], which is prior to the loss of regenerative capacity at P7 [4]. We demonstrate that CUGBP1 inactivation impairs neonatal cardiac regeneration at P1, in turn, replenishment of CUGBP1 levels prolong regenerative potential. These results indicate that postnatal CUGBP1 downregulation is indeed of crucial for the impaired regenerative ability of the heart after birth.

Whereas, when CUGBP1 is re-employed in the adult heart leading to dilated cardiomyopathy [9]. With respect to cardiac hypertrophy, a fetal gene reprogramming process, CUGBP1 is upregulated [15]. All these evidences indicate that keeping the proper expression level of CUGBP1 at the right time is important. Currently, in our study, we prolong regenerative potential up to P14 and none persistent impairments of the heart were observed post transient CUGBP1 expression manipulation. Following the further study efforts for CUGBP1-associated time window extending of regeneration ability in mammalian heart, the potential abnormalities caused by replenishment of CUGBP1 must study simultaneously.

### CUGBP1 is a critical regulator for cardiomyocyte proliferation

Proliferation of cardiomyocytes was mainly responsible for cardiac regeneration in neonatal mice and zebrafish [16]. Many previous studies have shown that CUGBP1 is involved in multiple types of cell proliferation [17–20]. More importantly, CUGBP1 plays a key role in liver injury repair and regeneration [20, 21]. Previously, we identified that CUGBP1 promotes the angiogenesis on acute myocardial infarction [22]. Meanwhile, HO-1, an important regulator of cell proliferation, was downregulated by CUGBP1 [15].

In current study, we found CUGBP1 markedly promoted cardiomyocyte proliferation and improved heart regeneration. Upon replenishment of CUGBP1 levels, we found that Sfrp5 and Wif1, Wnt inhibitory factors, were significantly downregulated. Furthermore, by using IWR-1, a selective Wnt inhibitor, the increased Ki67 levels upon CUGBP1 over-expression was significantly reduced. CUGBP1 mediates the pro-proliferative effect by activated the Wnt/β-catenin signaling pathway and promotes cardiomyocyte proliferation. Similarly, zhao and his colleagues showed that Notch signaling supports cardiomyocyte proliferation by reduced levels of Wnt antagonists and dampening Wnt activity during zebrafish heart regeneration [23]. Taken together, our findings provide the evidence to support a novel role of CUGBP1 as an important regulator for cardiomyocytes proliferation.

### CUGBP1-Wnt/β-catenin-GATA4 regulatory axis

Wnt/β-catenin signaling and GATA4 play crucial roles in heart development [7, 24]. There is increasing evidences indicate that Wnt/β-catenin signaling is involved in cardio-regenerative pathway driving cardiomyocyte proliferation [8]. The loss of GATA4 expression markedly impaired cardiac regeneration in zebrafish and mice [13, 25, 26].

During postnatal stage, the CUGBP1, GATA4 protein levels and Wnt/β-catenin signaling activity is downregulated after birth [6, 13, 27], accompanied by dramatically reduced heart regenerative capacity [4].

CUGBP1 levels was rapidly decreased after birth at P6 and GATA4 is strongly reduced at P7 [6, 13]. Wnt/β-catenin signaling is normally suppressed postnatally [27]. This pattern of expression hints a role of CUGBP1 as regulator of Wnt/β-catenin signaling and GATA4 during heart regeneration. Based on our results, we propose an integrated regulatory network, CUGBP1-Wnt/β-catenin-GATA4 regulatory axis, for heart regeneration. Current research provides first novel insight that the Wnt/β-catenin signaling and GATA4 involve in the CUGBP1 modulated neonatal heart regeneration.

The previous study has shown that GATA4 levels in human adipose stem cells significantly increased with activation of Wnt/β-catenin signaling [28]. Simultaneously, Duan and his colleagues showed that Wnt3 regulates axon regeneration by downregulation of Gata4 [29]. Whereas, we observe no significant difference in Wnt3a expression upon CUGBP1 over-expression (Supplementary Fig. S3). IWR-1 inhibits WNT-induced nuclear β-catenin accumulation, thus leading to proteasomal degradation of this protein [30]. We show that IWR-1 treatment dismisses CUGBP1-dependent regulation of GATA4. These results indicate that activation of Wnt-β-catenin signaling cascade by CUGBP1 is account for the regulation of GATA4 by CUGBP1 in this circumstance. CUGBP1 regulates gene expression at multiple steps of alternative splicing, translation, and mRNA degradation [31]. Therefore, it is necessary to conduct more in-depth research to address CUGBP1-Wnt/β-catenin-GATA4 regulatory axis.

Collectively, we demonstrate that cardiac inactivation of CUGBP1 impairs neonatal cardiac regeneration, in turn, replenishment of CUGBP1 levels prolong regenerative potential. Our study extends the time window of regeneration ability of the mammalian heart up to 14 days after birth. This research provides novel insights of the CUGBP1-Wnt/β-catenin-GATA4 regulatory axis involving in the neonatal heart regeneration. As conclusion, our findings support CUGBP1 as a key factor promoting post-injury cardiac regeneration and provide a potential therapeutic method for heart disease though further studies need to be conducted regarding the detailed mechanism.

## Methods

### Expression constructs

The CUGBP1, dominant-negative CELF (CELF∆) and DsRed cDNA sequences were amplified from the pcDNA3.1-hCUGBP1, pMHC-NLSCELF and pDsRed2-N1 vectors, respectively. The digested PCR fragments of CUGBP1, CELF∆ and DsRed were ligated into pShuttle-CMV to generate the pShuttle-CMV-DsRed-CUGBP1/CELF∆ expression plasmid. All obtained constructs were sequence-verified.

The cardiac tissue-specific dominant-negative transgene MHC-CELF△ plasmid provided by Thomas A Cooper (Department of Pathology, Baylor College of Medicine, Houston, TX) [10]. The nucleus-restricted dominant-negative CELF protein NLSCELF△ was created by insertion of the strong nuclear localization signal from the simian virus 40 large T antigen in frame between the N-terminal Xpress epitope tag and the truncated CELF4 open reading frame of the previously described CELF△ [11].

### Adenovirus generation

Adenovirus particles were produced following the manufacturer’s protocol (AdEasy™ XL Adenoviral Vector System, Stratagene, USA). The recombinant adenovirus plasmids were transfected into AD-293 cells by using Lipofectamine^TM^ 2000 (Invitrogen, Carlsbad, CA, USA). All the adenoviruses generated were purified by CsCl gradient ultracentrifuge and desalted using a Sepharose CL-4B column (GE Healthcare, Waukesha, WI, USA), and the viral concentrations were determined.

### Animal model

The use of animals and all experimental procedures were approved by the Institutional Animal Use and Care Committee of Institute of Biophysics at Chinese Academy of Sciences and complied with National Institute of Health guidelines. Timed-pregnant ICR/CD-1 mice (Beijing Vital River Laboratory Animal Technology Co., Ltd.) were used to deliver pups for neonatal surgical procedures. Investigators were blinded to the animals during the outcome assessments.

Apical resection was performed on neonatal mice at postnatal days as described previously [4]. Neonatal mice were anaesthetized by cooling on an ice bed for 2 min that do not require anesthesia agent. After skin incision, lateral thoracotomy at the third intercostal space was performed by blunt dissection of the intercostal muscles. Adenovirus particles were slowly injected at multiple locations in the LV wall with the ultra-fine needle insulin syringe(U-40, Becton, Dickinson and Company). A total of 50–75 μL adenovirus (4–6 × 10^10^ particles) containing CUGBP1/CUGBP dominant-negative protein (CUGBP∆) or control adenoviral vectors were delivered to the heart. Following the administration of adenovirus, the mice were subjected to apical resection or sham operation.

### Quantitative real-time PCR

Total RNA was extracted from heart tissue with Trizol reagent (Invitrogen, USA), and cDNA was synthesized from total RNA (2 μg) with the PrimeScript^TM^ RT Reagent Kit with gDNA Eraser (Takara Bio Inc, Japan). qPCR was performed on a Corbett Rotor-Gene 6600 QPCR system machine (QIAGEN) with the SYBR Green PCR kit (Applied Biological Materials, ABM) using a three-step protocol (denaturation at 95 °C for 30 s, annealing at 60 °C for 30 s, and extension at 72 °C for 30 s for 35 cycles). All samples were amplified in triplicate, and the mean was obtained for further calculations. Relative mRNA expression of target genes was normalized to β-actin and represented as fold change vs. control. Primers used in quantitative PCR are shown in Table 1.

**Table 1:** List of primers used in this study.

Name	Sequence (5’-3’)
Sfrp5-qPCR-F	GCACTGCCACAAGTTCCCCC
Sfrp5-qPCR-R	TCTGTTCCATGAGGCCATCAG
Wif1-qPCR-F	ACAAGTGCCAGTGTCGAGAG
Wif1-qPCR-R	AAATTCAGGCCGGCGTTCTA
Actin-F	GGACTGTTACTGAGCTGCGTT
Actin-R	CGCCTTCACCGTTCCAGTT
Sfrp5-RIP-PCR-F	CTGTGCCTTGCCTCGCCCTC
Sfrp5-RIP-PCR-R	CTCTGCCTGTTTTCTCAGAC
Wif1-RIP-PCR-F	CTGAAACGGTTCGAGTTACG
Wif1-RIP-PCR-R	TCTGTTCCCCCCTCAAAACTG
GATA4-RIP-PCR-F	CAAATACCTTTCCAGGTGGAG
GATA4-RIP-PCR-R	GAGAGAGCTTGGCTTGGCAG

### Western blot analysis

Heart tissues were homogenized and lysed in RIPA lysis buffer (Beyotime Bio Co, China). Proteins were separated by 12% SDS/PAGE, transferred to PVDF membranes (Millipore, USA), blocked and incubated with antibodies. Final detection was performed using enhanced chemiluminescence detection solutions 1 and 2 (1:1) (ECL; Millipore). The relative protein expression level was analyzed by densitometry using ImageJ software (Bethesda, Maryland, USA). Primary antibodies used in this study were as follows: anti-CUGBP1 (sc-20003, Santa Cruz Biotechnology, USA), anti-Xpress (R910-25, Thermo, USA), β-actin (AA128-1, Beyotime, China) or GAPDH (AG019-1, Beyotime, China) were detected as control.

### Histology (Hematoxylin&eosin and Masson’s trichrome staining)

Following the isolation from mice, heart tissues were fixed in 4% paraformaldehyde on a shaking table overnight at room temperature. Heart samples then were moved to 70% ethanol for paraffin embedding and sectioning (5 μm) later. Haematoxylin and eosin (H&E) staining and Masson’s trichrome staining were performed according to standard procedures.

### Transthoracic echocardiography

Mice were anesthetized with isoflurane, and then subjected to transthoracic echocardiography using the Visual Sonics Vevo® 2100 Imaging System, equipped with a 40 MHz mouse ultrasound probe. Ejection fraction (EF) and fractional shortening (FS) were used to calculate left ventricular systolic function based on end diastolic and end systolic dimensions obtained by M-mode ultrasound. Echocardiography was performed on 3–9 mice in each group.

### Immunofluorescence analysis

Following deparaffinization and high-pressure antigen retrieval with 10 mM sodium citrate buffer (PH 6.0) in boiling water for 2 min, paraffin sections were blocked with 10% goat serum (ZLI-9022, ZSGB-BIO, China) for 1 h. Primary antibodies against PH3 (ab177218, abcam), ki67 (ab16667, abcam), CD31 (ab28364, abcam) and cTnI (66376-1-Ig, proteintech) were incubated overnight at 4 °C. Then, resections were washed with phosphate buffered saline (PBS) three times and incubated with corresponding secondary antibodies conjugated to Alexa Fluor 488 and Alexa Fluor 594 (Invitrogen) for 1 h. Nuclei were counterstained by DAPI (Vector Labs) for 30 min. Fluorescence was examined with a Laser scanning Confocal Microscopy (Nikon). All staining was performed on 3–6 hearts each group and 3 slices were obtained from each heart.

### Isolation and culture of neonatal rat ventricular myocytes (NRVMs)

Primary NRVMs were isolated from 1-day-old Sprague-Dawley rats using a modified collagenase dissociation protocol. Briefly, the hearts were removed from the pups immediately after euthanasia, the ventricles were excised and minced in buffer (137 mM NaCl, 5.4 mM KCl, 1.2 mM NaH_2_PO_4_, 1.2 mMMgCl_2_, 20 mM Hepes, 10 mM Glucose, 10 mM 2,3-butanedione monoxime (BDM), 10 mM Taurine, pH 7.36), and then cardiomyocytes were dissociated with collagenase II (1 mg/mL, 17101-015, LABLEAD). These cells were then resuspended in Dulbecco’s modified Eagle medium (DMEM; (GIBCO)) supplemented with 10% fetal bovine serum (FBS; (Hyclone)), and plated in culture dishes for 40 min for differential adhesion. Cardiomyocytes were purified by using Percoll density gradient centrifugation [32] and plated in different culture dishes coated with gelatin. On the following day, the medium was changed to remove dead cells.

### Transcriptomic analysis

Hearts at 7 days after apical reaction and CUGBP1 over-expression at P8 from mice were rapidly excised, placed in room temperature PBS to evacuate blood, and then store in −80 °C. Total RNA was extracted with Trizol reagent (Invitrogen,USA). Transcriptome sequencing was performed by the Beijing Genomics Institute (BGI) using the the Illumina HiSeq-PE150 Platform. The sample quality was tested in Agilent 2100 Bioanalyzer (Agilent RNA 6000 nano kit) to be up to standard to construct RNAseq sequencing libraries. We sequenced six samples on Illumina HiSeq Platform in total and generated about 6.71 Gb per sample library. Data were processed as previously described [33].

### RNA immunoprecipitation

NRVMs were maintained in DMEM (Gibco) supplemented with 10% FBS and antibiotics, at 37 °C and 5% CO_2_. RIP experiment was performed as previously described [34]. Briefly, antibody to CUGBP1 (Santa Cruz Biotechnology, cat# sc-20003), and IgG (Santa Cruz Biotechnology, cat# sc-2025) was added to supernatant for immunoprecipitation (IP) and mock group, respectively. Twenty microliters of protein A/G beads (Santa Cruz Biotechnology, cat# sc-2003) were added in the supernatant following the adding of antibody and IgG. After washing, the beads were resuspended in 1 mL of Trizol to isolate coprecipitated RNAs according to the manufacturer’s instruction (Invitrogen).

### Statistical analysis

Significant differences were determined between two groups using Student’s *t*-test or ANOVA among multiple groups for independent samples as indicated in the figure legends. Data are presented as the mean ± SEM from at least three biological replicates. Statistical analysis was performed using GraphPad Prism 6. *p*-value < 0.05 was considered statistically significant. No randomization was used.

## Supplementary information

Supplementary figure legends

Fig. S1. Recombinant adenoviral gene transfer CUGBP1 or CUGBP∆ into neonatal hearts.

Fig. S2. RNA sequencing analysis.

Fig. S3. The influence of Wnt3a levels upon CUGBP1 over-expression

## Data Availability

All data generated or analyzed during this study are included in this published article and its Supplementary Information files.
