# Supplementary material for: CUGBP1, a crucial factor for heart regeneration in mice
Source: Cell Death Dis. 2022 Feb 8;13(2):120. doi: 10.1038/s41419-022-04570-w (PMC8825809; doi:10.1038/s41419-022-04570-w)
Supplement: Supplementary file 1 — Supplementary figure legends [file 41419_2022_4570_MOESM1_ESM.docx]

**Supplementary figure legends**

**Fig. S1. Recombinant adenoviral gene transfer CUGBP1 or CUGBP∆ into neonatal hearts.**

**A** The recombinant adenovirus was used to introduce CUGBP1 or CUGBP∆ into neonatal hearts. Seven days after the adenovirus injection, the whole heart was collected and photographed under fluorescent microscope (Left, bright field; Middle, red fluorescence induced by mercury vapor lamp; Right, the combination of left two figures). **B** Western blotting was performed to detect the expression of CUGBP1 and CUGBP∆. Left panel: CUGBP1 was overexpressed by adenovirus injection into the heart of 8-day old mice and resection was performed at the same time. The expression of CUGBP1 in the heart was detected by Western blot at 7 days after surgery. Right panel: One day after cardiac injection of CUGBP1∆ adenovirus, apical resection was performed in mice. The expression of X-press tagged CELF∆ in the heart was detected by Western blot 7 days after surgery. **C, D** Ultrasound assessment of mice hearts in 21 days post adenovirus injection. Data are the mean ± SEM (n= 4-6).

**Fig. S2. RNA sequencing analysis**.

RNA-sequencing was performed from the hearts of AdDsRed and AdCUGBP1 mice (n=3 for each group). **A** The Volcano plot shows differently expressed genes (p-adj<0.05, fold-change ≥2) in AdDsRed and AdCUGBP1 mice. Red color indicates up-regulated, while blue represents down regulated genes. Black color indicates unchanged expression levels. **B** The heat map shows significantly dysregulated genes (down-regulated: 555, up-regulated 743 genes) Red color represents up-regulated genes, while the blue color shows the down-regulated. **C** Dot plots shows top 10 GO biological process, **D** cellular component, **E** molecular function and **F** KEGG pathways that were enriched from differently expressed genes.

**Fig. S3. The influence of Wnt3a levels upon CUGBP1 over-expression**

**A** Western blotting was performed to detect the expression of Wnt3a upon CUGBP1 over-expression. **B** Western blot bands were quantified by densitometry and normalized to actin. Data are the mean ± SEM (n= 3).
