# Supplementary figures and images for: CUGBP1, a crucial factor for heart regeneration in mice
Source: Cell Death Dis. 2022 Feb 8;13(2):120. doi: 10.1038/s41419-022-04570-w (PMC8825809; doi:10.1038/s41419-022-04570-w)

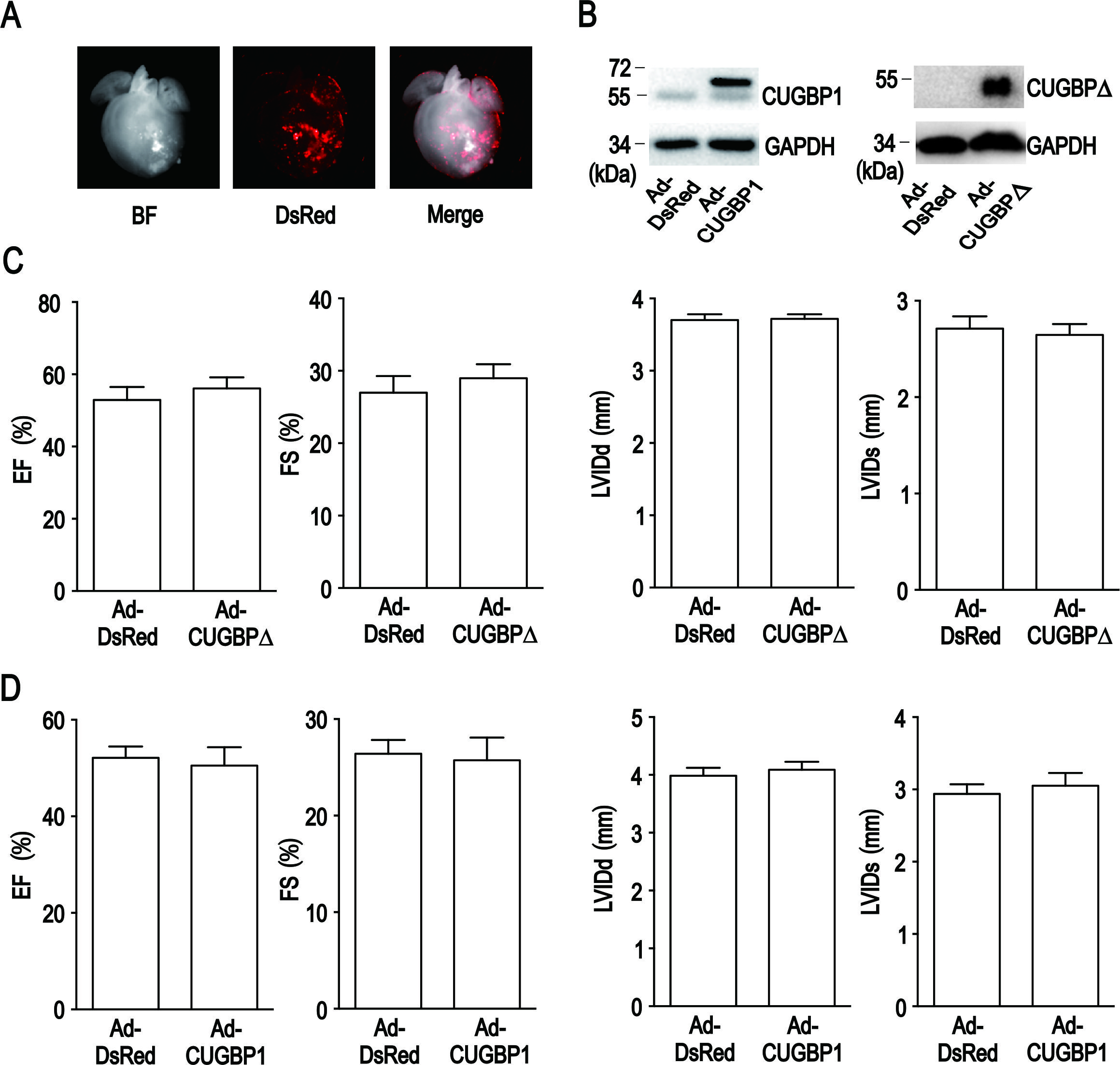

Supplement: Supplementary file 2 — Fig. S1. Recombinant adenoviral gene transfer CUGBP1 or CUGBP∆ into neonatal hearts. [file 41419_2022_4570_MOESM2_ESM.jpg]

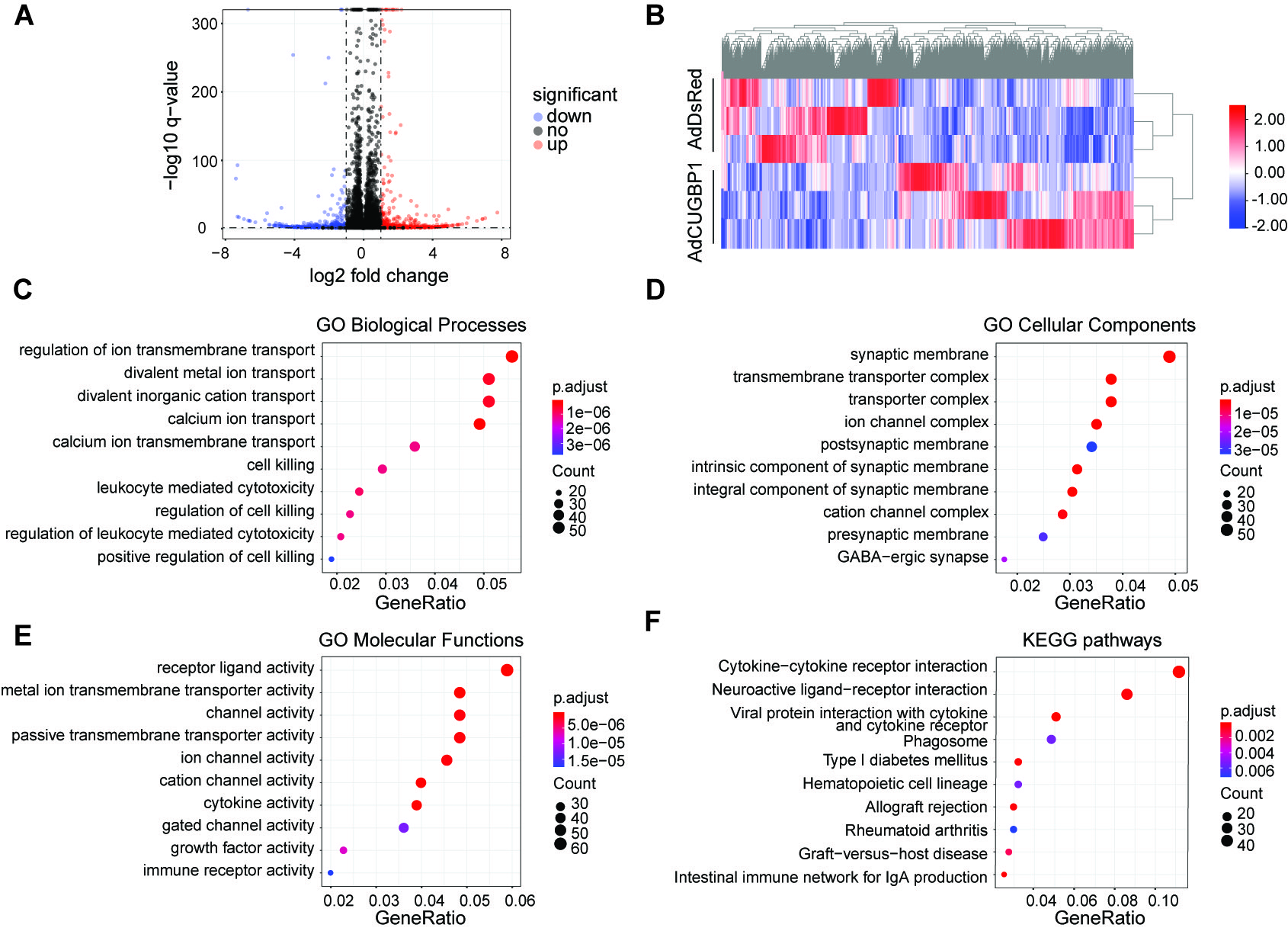

Supplement: Supplementary file 3 — Fig. S2. RNA sequencing analysis. [file 41419_2022_4570_MOESM3_ESM.jpg]

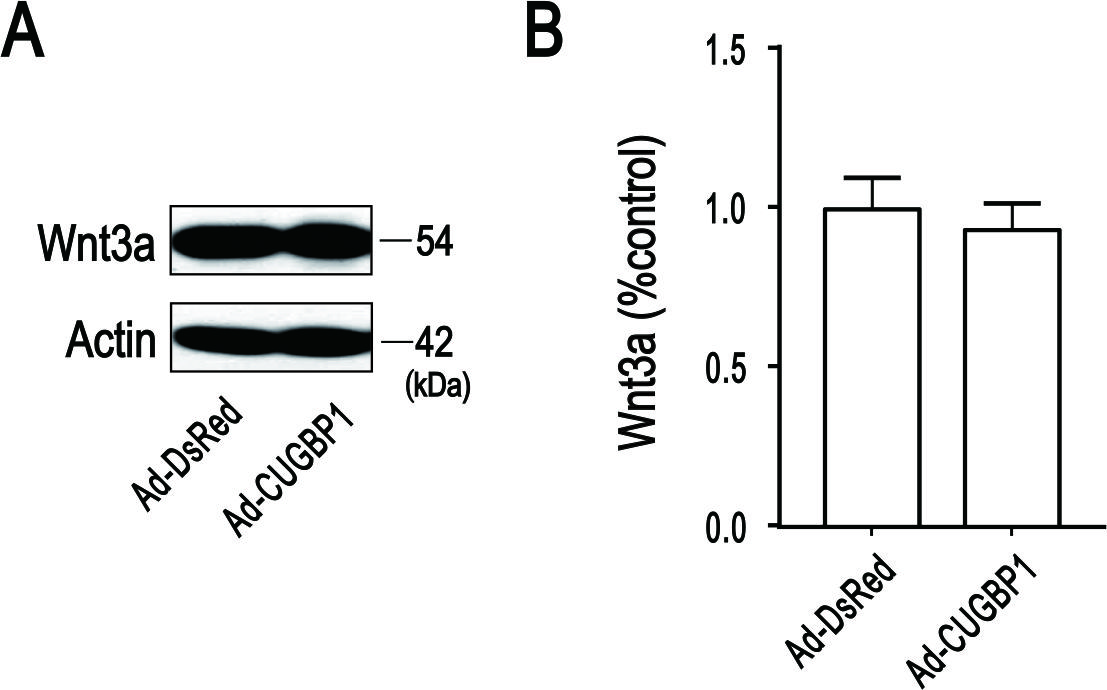

Supplement: Supplementary file 4 — Fig. S3. The influence of Wnt3a levels upon CUGBP1 over-expression [file 41419_2022_4570_MOESM4_ESM.jpg]
